# The application of flow to an ultrasonic horn system: Phenol degradation and sonoluminescence

**DOI:** 10.1016/j.ultsonch.2020.105373

**Published:** 2020-10-20

**Authors:** Richard James Wood, Audrey Bertin, Judy Lee, Madeleine J. Bussemaker

**Affiliations:** Department of Chemical and Process Engineering, University of Surrey, Guildford, Surrey GU2 7XH, United Kingdom

## Abstract

Sonochemical (SC) processes can be increased with the application of fluid flow due to changes in bubble characteristics. In this work, a novel flow through set-up was applied to an ultrasonic horn system to investigate the effects of flow on the degradation of phenol. KI dosimetry and sonochemiluminescence (SCL) were also analysed, under the same conditions, to provide comparison of degradation to other SC processes. Further, sonoluminescence (SL) in water and different concentrations of potassium iodide (KI) and phenol solutions was studied to determine the effect of flow on processes inside the bubble that result in SL. The degradation of 0.1 mM phenol solutions, KI dosimetry and SL from phenol (0.1, 20 and 60 mM) and KI (0.1, 1 and 2 M) solutions were analysed under flow rates of 0, 24, 228 and 626 mL/min. For an ultrasonic horn system, all flow rates could augment phenol degradation beyond that of the systems without flow. At the lowest applied power, the amount of degradation was significantly increased with flow, becoming greater than degradation observed at the highest power. A strong correlation between phenol degradation and SC processes indicated that degradation followed an oxidative process. SL intensity from water, KI, and phenol solutions could also be increased with flow beyond the no flow system. For water this occurred most readily at higher powers, then for the solutes there was varied behaviour dependent upon the solute concentration. It was theorised that flow may increase the transfer of radical species to solution to enhance SC processes. An increase in SL, with flow, indicates that flow is acting to change the properties of the bubbles and/or the bubble field such that the active bubbles present collapse with greater total intensity.

## Highlights

• Ultrasonic horn degradation of phenol augmented with fluid flow.
• Similar degradation trends to other sonochemical oxidation processes.
• Reduction in collapse intensity reduces contribution from pyrolytic degradation.
• Augmentation of sonoluminescence from phenol and salt solutions with flow.

## 1 Introduction

In previous work the degradation of phenol was analysed, under fluid flow, using ultrasonic plate transducers of frequency; 44, 300 and 1000 kHz [1]. At 300 kHz there was very close correlation between potassium iodide (KI) dosimetry and phenol degradation indicating an oxidation process, in agreement with observations from other authors at 487 kHz [2]. At 44 and 1000 kHz, although oxidation of phenol was the primary degradation mechanism, there was some disparity between the processes. However, fluid flow, under certain conditions, was able to increase phenol degradation at all applied frequencies for the plate system [1]. The effects of flow on sonoluminescence (SL) from phenol and salt solutions was also studied for a plate transducer at low frequency (44 kHz) [1], [3]. Fluid flow produced similar characteristics to streaming and, as observed by other authors [4], [5], could significantly enhance SL emission. An ultrasonic horn system produces a different cavitation environment, compared to an ultrasonic plate. High-frequency plate systems are better known for higher radical production, however large scale, low frequency systems are often proposed (e.g. for wastewaters [6], steel corrosion [7] and for increased efficiency [8]). Therefore, it is important to determine the effects of flow with respect to changes in sonochemical (SC) characteristics and degradation processes for a horn-type system.

An ultrasonic horn consists of both travelling and standing waves, as with a plate transducer, however the travelling wave component for the horn almost entirely dominates the system, especially at higher powers [9], [10]. The region of active cavitation, for an ultrasonic horn, tends to be localised near the horn tip [11]. Turbulent kinetic energy measurements which indicate regions of energy dissipation, for a 2 L reactor, showed that 85% of energy from the horn is dissipated close to the horn tip in 2% of the total volume of solution [12]. The primary and secondary Bjerknes forces causes bubbles to congregate in this region [13], with bubble–bubble interactions significantly reducing bubble growth [14]. The formation of a conical bubble structure and a thin layer of bubbles close to the tip acts to impede the propagation of the sound field [13]. The thickness of the layer increases with the pressure amplitude and may result in a strong standing wave between the horn and the bubble layer, however the layer’s non-linear thickness distorts the formation [13].

This work considered the degradation of phenol using a 20 kHz ultrasonic horn under fluid flow conditions. KI dosimetry and sonochemiluminescence (SCL) from luminol solution were studied under the same conditions to determine any similarities with these other SC processes . The horn represents a different ultrasound system, than a plate system where fluid flow may increase sono-process efficiency that can inform design and development of larger scale reactors. SL from water, phenol and KI solutions under horn sonication and flow conditions were also studied since changes in SL were previously observed under flow conditions for a low frequency (44 kHz) ultrasonic plate [1], [3]. To the authors’ knowledge this is the first time a flow system has been applied to an ultrasonic horn system to aid in the understanding of fluid flow on these processes.

## 2 Experimental

### 2.1 Reactor configuration

**Fig. 1:**
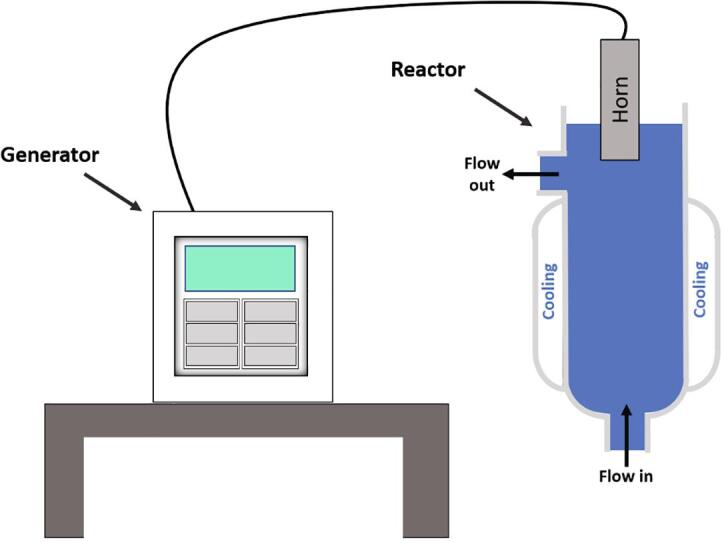
Experimental setup (not to scale). The ultrasonic generator is connected to the ultrasonic horn which is inserted into the top of the vessel for operation at 20 kHz. A peristaltic pump circulates the sample flow and an open bath circulation system circulates the cooling flow (not shown).

A schematic of the experimental setup is shown in Fig. 1. The glass reactor was custom designed and then made by the University of Southampton (UK) Scientific Glassblowing Service. The inner diameter and height of the vessel measured 30 mm and 140 mm, respectively. Water at room temperature was circulated through the vessel’s cooling jacket and maintained by a Cole-Palmer PolyScience MX 20L, open bath circulation system. The signal to the Langevin type transducer (horn) was sent from a Fisher Scientific FB-705 generator and the horn tip was 12.7 mm in diameter. A Watson Marlow 313S peristaltic pump was utilised in conjunction with chemically resistant TYGON® tubing to achieve flow rates of 0, 24, 226 and 626 mL/min. The volume of samples were 62 mL without flow and 72 mL for the flow systems to account for solution in the tubing and ensuring a constant liquid height of 90 mm (from flow inlet) for all experiments. The flow was applied in a closed loop such that aeration of solution would be minimised. The frequency of operation of the ultrasonic horn was 20 kHz and sonication time was 14 min, in line with previous work [1], [3], [15]. Experiments were conducted at room temperature (25 ± 3) °C and for a minimum of 3 times each.

### 2.2 Methodology

#### 2.2.1 Materials

All chemicals; potassium iodide (KI), ammonium chloride (NH_4_Cl), ammonia hydroxide solution (NH_4_OH), potassium hexacyanoferrate (III) (K_3_Fe(CN)_6_), 4-aminoantipyrine (C_11_H_13_N_3_O), phenol (C_6_H_5_OH) were acquired from Sigma-Aldrich®. All solutions were made using distilled water from an Elix Essential 3 (UV) Type 2 operating at 14.5 mΩ cm.

#### 2.2.2 Calorimetry

Calorimetry was used to determine the ultrasonic power in solution [16], these values are shown in Table 1. Despite an increase in the applied power from 10 to 40 W at the generator, the calorimetric power is comparable for 10 and 20 W, and 30 and 40 W. This demonstrates the difficulty in acquiring an accurate value for the power in solution, especially in a more turbulent system as is observed for the low frequency horn. Set powers have been presented in the results and discussion for simplicity.

**Table 1:** Calorimetric powers for the 20 kHz ultrasonic horn at 10, 20, 30 and 40 W.

Applied Power (W)	Calorimetric Power (W)
10	8.5 ± 0.3
20	8.5 ± 0.5
30	10.5 ± 1.0
40	10.6 ± 0.6

#### 2.2.3 KI dosimetry

KI solution (0.1 M) was used to give a measure of sonochemical activity via the oxidation of iodide ions to the triiodide ion ($I_{3}^{-}$) that absorbs spectrophotometrically at 355 nm [16]. More selective radical dosimeters are available [17], however KI represents a simple and widely accepted method to determine overall sonochemistry. $I_{3}^{-}$ concentration was determined by spectrophotometric analysis (Thermo Scientific Evolution 201 UV–visible spectrometer) using a molar absorptivity coefficient of 26,303 L/mol cm [16].

#### 2.2.4 Phenol analysis

The method for phenol analysis is presented in previous work [1]. It employs the 4-aminoantipyrine (4-AAP) method where phenol couples with 4-AAP in an alkaline solution (potassium hexacyanoferrate (III) at pH 7.9) to form a coloured antipyrine complex (AMPH), detected by UV spectrometry at 510 nm [18], [19].

#### 2.2.5 Sonochemiluminescence/sonoluminescence image analysis

SCL and SL analysis was undertaken to measure the intensity of sonochemical/ionising reactions. SCL was performed using a 1 mM luminol (5-amino-2,3-dihydro-1,4-phthalazinedione) and 0.1 M NaOH (pH 13) solution [20]. An ANDOR iXon3 EMCCD camera and software was used to capture and quantify luminescence emission. The camera operated at −70 °C and applied an EM gain level/exposure time (seconds) of 50/40 for SL and 4/4 for SCL.

## 3 Results and discussion

### 3.1 The effect of flow on phenol degradation, SCL and iodide dosimetry

**Fig. 2:**
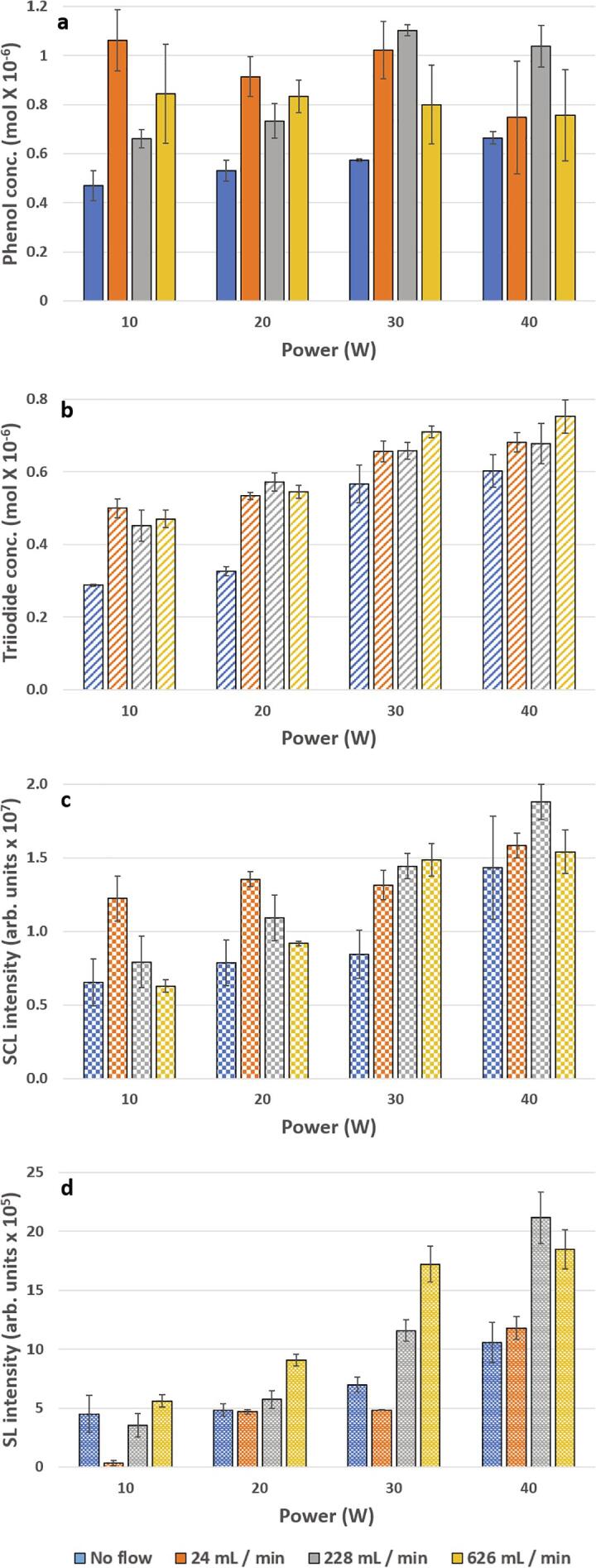
Different measures of active cavitation: (a) Phenol degradation (b) triiodide production, (c) SCL intensity from luminol solutions and (d) SL intensity from water at different powers and for flow rates of no flow (blue), 24 mL/min (orange), 228 mL/min (grey) and 626 mL/min (yellow). (For interpretation of the references to colour in this figure legend, the reader is referred to the web version of this article.)

**Fig. 3:**
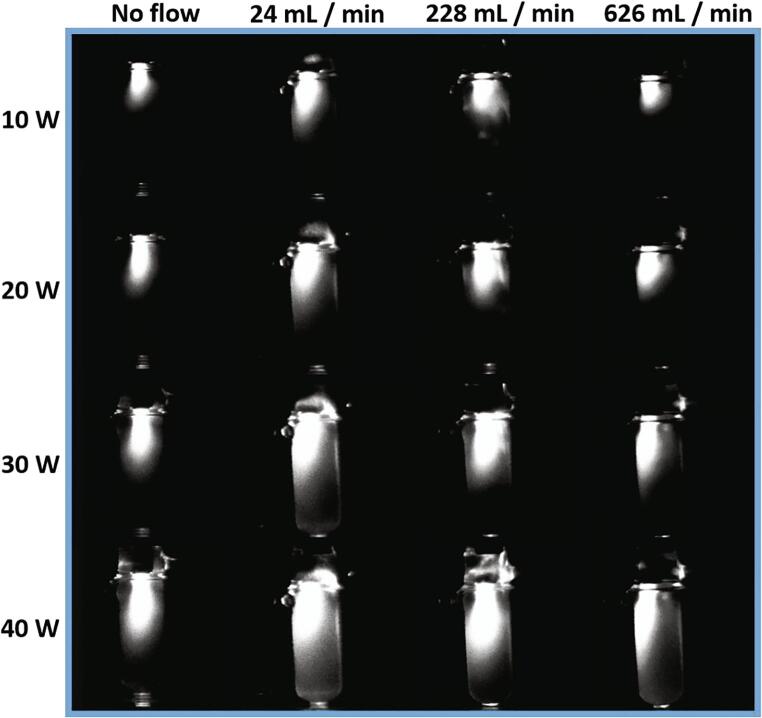
SCL intensity distribution at 44 kHz for no flow, 24, 228 and 626 mL/min systems at 10, 20, 30 and 40 W.

External fluid flow can significantly enhance phenol degradation, triiodide production and SCL intensity as shown in Fig. 2(a – c). For a flow rate of 24 mL/min at 10, 20 and 30 W, phenol degradation increases from no flow by 126, 72 and 78%, respectively (Fig. 2a). At the same flow rate, from no flow, there are 74, 63, 16% increases in the dosimetry (Fig. 2b), and 88, 72, 56% increases in SCL intensity for 10, 20 and 30 W, respectively (Fig. 2c). The SCL distribution shows an increase in width and length for 24 mL/min in comparison to no flow across the power range (Fig. 3).

As flow rate is increased, at each power, there are some similarities between phenol degradation and SCL (Fig. 2a, c). For example, at 10 W, after the initial increase from no flow at 24 mL/min, from 24 to 228 mL/min there are 61 and 35% decreases in phenol degradation and SCL respectively, then there is no change from 228 to 626 mL/min. In this instance the SCL distribution reduces in length (from 24 to 228 mL/min) (Fig. 3). The dosimetry also shows some correlation to phenol degradation; where all flow rates at 10, 20 and 30 W increase the SC processes beyond no flow (Fig. 2a, b). Although ultrasonic plate and horn systems have different fields of bubble activity, the behaviour for the horn is consistent with several theorised flow effects, namely; a contribution to bubble transience [21] which may aid in transfer of radical species to solution [20], [22], reducing coalescence of bubbles to inactive size [5] or breaking apart bubble clusters which impede the sound field [4]. For SCL, where activity is expanded, there may also be some contribution from streamers [23] (previous authors have shown streams of bubbles from micropits can produce OH radicals and expand the SCL region [24]) as they are projected away from the horn tip.

At the highest applied power (40 W), only a flow rate of 228 mL/min is able to increase phenol degradation beyond no flow (by 36%) (Fig. 2a). This suggests some limitation to the applied flow and could imply that a flow rate of 24 mL/min is no longer sufficient to affect the system due to an increase in the field strength/bubble impedance [13]. Then, at 626 mL/min (30 and 40 W), as for the 30 W phenol and 40 W SCL systems, there is a decrease in phenol degradation from 228 to 626 mL/min here implying flows effects have become unfavourable for the SC processes. It should be noted that KI dosimetry, unlike phenol degradation and SCL, does not show a decrease from 228 to 626 mL/min (Fig. 2b). Similar observations were made in previous work, for an ultrasonic plate, where dosimetry yields could be increased or show no reduction under higher applied flow rates, yet SCL intensity decreased [15].

Thus, the results show that the degradation of phenol can be augmented with fluid flow, to a point, for an ultrasonic horn system. The observed correlations between phenol degradation, dosimetry and SCL (intensity and distribution) implies that phenol is degraded via an oxidation process dominated by OH radical release, as previously observed [2], [25], [26].

### 3.2 The effect of flow on SL

**Fig. 4:**
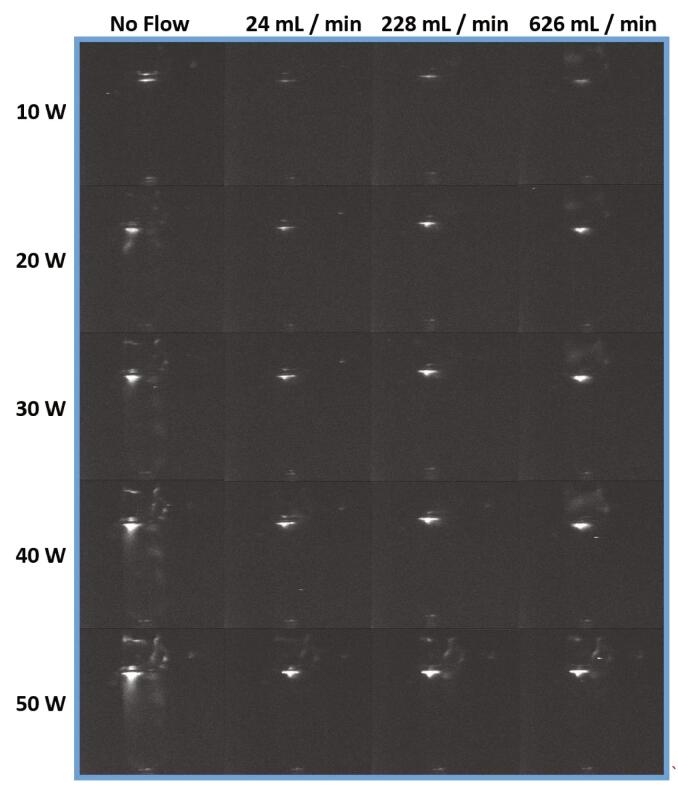
SL intensity distribution from water at 20 kHz for no flow, 24, 228 and 626 mL/min systems at 10, 20, 30, 40 and 50 W.

At 10 and 30 W, SL from water is significantly decreased from no flow by 93 and 31% at 24 mL/min (Fig. 2d). The SL distribution, although difficult to determine due to the low intensity of light, shows some reduction in activity towards the surface of solution (Fig. 4). As power is increased the higher flow rates (228 and 626 mL/min) show significant increase in SL from no flow (Fig. 2d). The SL images show that although the distribution is decreased at these higher flow rates there is still activity near the horn tip (Fig. 4). This indicates that SL intensity at the tip must have significantly increased in intensity in order to achieve a higher intensity measurement (not determinable by visual inspection of the images). Due to the complex nature of ultrasound systems, no two ultrasound systems will demonstrate exactly the same effects, this is especially true of plate and horn systems. However, it has been demonstrated here for the horn system that, similar to a plate system, fluid flow is able to change the characteristic of bubbles/the bubble field, showing both decreases and increases in SL (from no flow). For SL, fluid flow has the potential to cause an increase in bubble transience [21] which can reduce the intensity of collapse [27] and the number of cycles for light emission due to earlier fragmentation [10]. Then, at higher powers the thickness of the attenuating bubble layer/clusters can increase, observed by other authors [13]. Here, flow may be contributing to their removal, allowing for greater dissipation of ultrasound energy into solution.

### 3.3 Implications of observed trends with applied fluid flow

The results have shown that flow can significantly enhance phenol degradation for an ultrasonic horn system. The correlation, under flow conditions, between phenol degradation, KI dosimetry and SCL indicate that phenol is degraded primarily via an oxidation process in these conditions. The effect of flow may be to increase the travelling wave of the system [21]. In turn, aiding in the transfer of radical species to solution as bubbles become more transient and prone to fragmentation [20]. However, the collapse intensity of the bubbles in this scenario will be significantly decreased [15], and so will any high temperature reactions inside the bubbles. Therefore, since radical production will be reduced [22], SC processes, such as phenol degradation, KI dosimetry and SCL, will depend heavily on the aforementioned fragmentation effects. Thus, for the SC processes of the ultrasonic horn system under flow, there may be a balance between an increase in bubble transience leading to transfer of radical species to solution, and a reduction in collapse intensity which produces fewer radicals.

For SL, flow, at low powers and flow rates shows limited effects, however as flow rate and powerare both increased, SL intensity is more readily augmented (Figure 2d), again indicating competing effects. This behaviour is consistent with flow removing bubbles from the attenuating bubble layer/clustering bubbles which may be increased at higher ultrasound powers, as suggested by other authors for a horn system [13]. Further work is suggested to directly image cavitation bubble behaviour under ultrasound horn conditions to elucidate the flow mechanisms that increase phenol degradation, other sonochemical processes and SL.

### 3.4 The effects of concentration and flow on SL from phenol and KI solutions

Thus far, the results have shown that sonochemical (SC) processes, and sonoluminescence (SL) in water, can be increased for an ultrasonic horn system under applied fluid flow. This confirms that flow can augment process for both ultrasonic horn and plate systems. In previous work, SL from phenol [1] and salt [3] solutions, for an ultrasonic plate system, could be increased under flow conditions. Thus, it is of interest to determine if SL can be similarly increased under flow for the horn system in the presence of organics/salts. As previously mentioned, SL from the horn system was very low in intensity and as such changes in the SL distributions (from the imagery) were difficult to determine. Therefore, only the changes in SL intensity are discussed, however the SL distributions have been presented in the Supplementary Information for completion.

#### 3.4.1 SL in phenol solutions

**Fig. 5:**
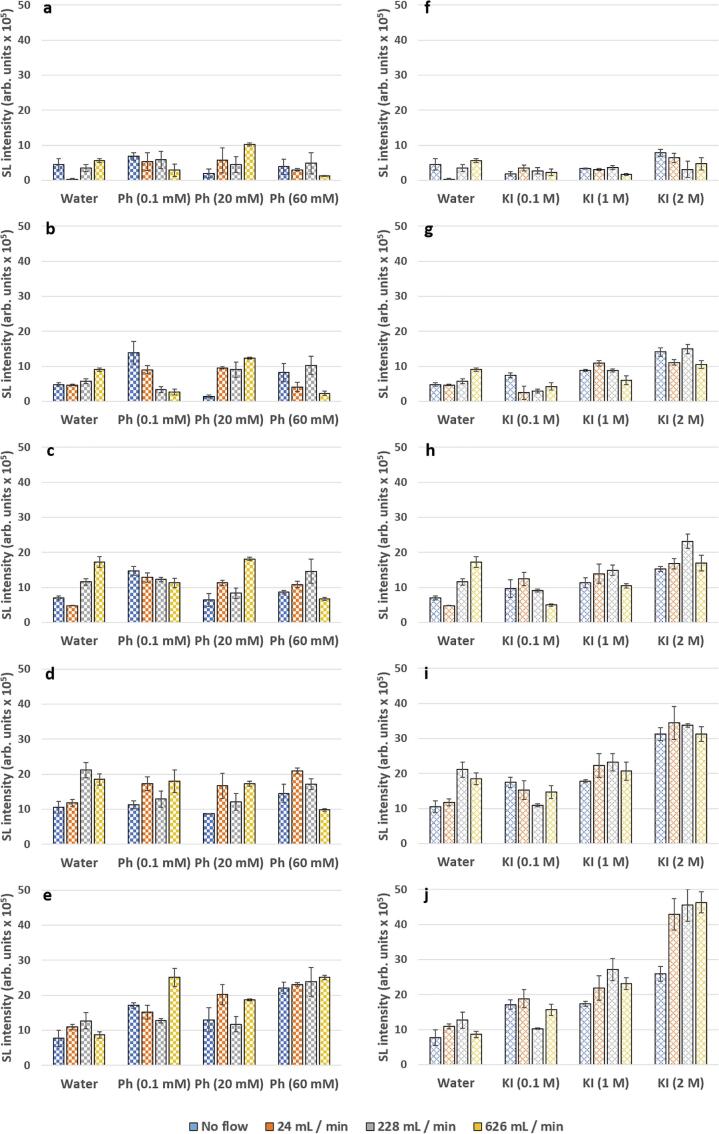
SL intensities at for phenol (0.1, 20 and 60 mM) (left) and KI (0.1, 1 and 2 M) (right) from 10 to 50 W (top to bottom in 10 W increments) for no flow (blue), 24 mL/min (orange), 228 mL/min (grey) and 626 mL/min (yellow). Water is included for comparison. (For interpretation of the references to colour in this figure legend, the reader is referred to the web version of this article.)

SL intensities from phenol solutions are presented in Fig. 5(a – e) where SL intensity values from 0.1 mM phenol solutions are greater than SL from water ~ 70% of the time. For example, for the system without flow at 30 W (Fig. 5c), SL from phenol shows an 110% increase in comparison to water. Since phenol can be considered a more volatile and polyatomic molecule than water, this result may be surprising as increased vaporisation of phenol into the bubbles may lead to ‘cushioning’ of the bubble collapse, and a reduction in SL [28]. Therefore, phenol itself is likely having some physical effect on the distribution/characteristics of the bubbles. Of note, is that the decreases in SL from phenol solutions, in comparison to water, only occur at the higher flow rates of 228 mL/min (20 and 40 W) (Fig. 5b, d) and 626 mL/min (10, 20 and 30 W) (Fig. 5a – c). For example, at 30 W 626 mL/min the SL from phenol is less than water by 34% (Fig. 5c). This shows similarity with previous work for an ultrasonic plate where bubbles were suggested to become more transient in nature with flow [21], allowing for an increase in diffusion/injection of solute into the bubbles [1]. Such effects can lead to a decrease in SL by reducing bubble collapse temperatures, and the production of excited species, that contribute to SL emission [29].

Before considering the application of fluid flow to the higher concentration phenol system, it is of use to consider how changes in phenol concentration, in the absence of flow, affect the system. For the phenol systems without flow, there was a general trend, across the power range, of an increase in SL in comparison to water at a concentration of 0.1 mM (Fig. 5a – e). This is followed by decrease at 20 mM, then an increase at 60 mM. For example, at 20 W, the 0.1 mM no flow, phenol system (Fig. 5b) shows a 187% increase in SL in comparison to water, which is then followed by a 90% decrease (20 mM) and 479% increase (60 mM). This shows very similar behaviour to the previously studied 44 kHz plate system under high power conditions without surface stabilisation [1]. The increases in SL between water and 0.1 mM phenol could be due to a reduction in coalescence. However, for such a low phenol concentration this behaviour may not be expected, and further work is required to elucidate the SL increase. Then, the decrease in SL from 0.1 to 20 Mm fits with an increase in surface concentration and associated quenching. Lastly, as surface concentration increases (60 mM) this may cause a reduction in coalescence to dominate over quenching effects, as observed for other organic solutes [29]. Since, the trends of the horn at lower powers match the trends of the plate at higher powers (where transience increases [30]), this implies that the horn represents a much more transient system, as expected [9], [11]. Therefore, quenching may occur more readily for the horn system via diffusion/injection mechanisms, as previously discussed. Thus, the phenol horn system without flow behaves very much like a low frequency plate system under more transient conditions at high powers.

External fluid flow can cause an increase in SL intensity from phenol solutions, particularly at higher powers or concentrations (Fig. 5a – e). For the 0.1 mM phenol solutions, an increase in flow at 10, 20 and 30 W shows a trend of decreasing SL (Fig. 5a – c). For example, from no flow to 626 mL/min there was a total decrease of 23% at 30 W (Fig. 5c). However, at 40 W flow rates of 24 and 228 mL/min increase SL from no flow by 54 and 14% (Fig. 5d), then at 50 W a flow rate of 626 mL/min shows a 40% increase (Fig. 5e). Similar observations are made for the 60 mM phenol solutions; at low power (10 and 20 W) SL intensity either decreases with flow, from no flow, or remains the same (Fig. 5a, b). However, at powers above 20 W flow leads to an increase in SL intensity beyond the no flow systems (Fig. 5c–e).

It was discussed previously (Section 3.2), where an increase in SL for water was observed at high powers and flow rates, that flow may contribute to the removal of attenuating/clustering bubbles to reduce attenuation on the sound field. At the lower powers, bubbles will likely be smaller in size and more susceptible to flow effects [15]. Thus, flow may act to both decrease the intensity of collapse and number of cycles for emission as the bubbles become more transient in nature [10], [21], [27]. This is supported by a general decrease in SL as flow increases for 0.1 and 60 mM systems. At 20 mM a different trend is observed; with, a general increase in SL from no flow at 24 mL/min, followed by a decrease at 228 mL/min, then a final increase at 626 mL/min. For example, at 50 W, SL shows a 55% increase (24 mL/min), 42% decrease (228 mL/min), then a 59% increase (626 mL/min) in SL intensity as flow is increased (Fig. 5e). This implies competing effects between an increase in phenol concentration, power and applied flow, all of which influence the characteristics of the bubbles and the resulting SL intensity. Therefore, for the phenol systems under horn sonication, applied flow can act to increase SL intensity, dependent upon power and phenol concentration.

#### 3.4.2 SL in KI solutions

In comparison to water, SL from 0.1 M KI solutions could be increased, most readily with an increase in power (Fig. 5f – j). However, as opposed to water, flow did not increase SL from the 0.1 M KI solutions indicating increased susceptibility to transient flow effects. An increase in concentration of KI from 0.1 to 2 M generally increases SL intensity without flow (Fig. 5f – j). This differs from the KI system of a 44 kHz plate where, in general, there was an increase in SL from water to 1 M [3] attributed to reduced gas concentration and bubble clustering [31], [32], followed by a decrease from 1 to 2 M proposed as a result of a reduction in collapse intensity of the smaller bubbles present in salt solutions [3]. Other authors have shown that, for an ultrasonic horn system and in general, active bubbles are localised close to the horn tip [13]. Therefore, this may imply that KI increases SL but only in instances where active bubbles become more localised and the intensity of collapse is not reduced by other factors.

For the 0.1 M KI system, flow did not increase SL intensity beyond no flow across the power and flow range, except at 10 W where there was an 86% increase at 24 mL/min (Fig. 5f – j). At 40 and 50 W there was a similar trend where a flow rate of 228 mL/min decreased SL intensity, followed by an increase at 626 mL/min (Fig. 5i, j). At 20 W, all SL values with flow were decreased from no flow, then at 30 W flow rates of 24 and 228 mL/min could remain comparable to no flow, followed by a 48% decrease at 626 mL/min (Fig. 5h). Since, flow in general leads to a decrease in SL intensity this suggests that the flow contributes to an increase in bubble transience [10], [21], [27]. However, an increase in SL intensity between flow rates at 40 and 50 W, as with previously discussed systems, implies some influence from flow on the system which negates any transience effects at higher powers. An increase in salt concentration to 1 M changes the system; in general, there is an initial increase in SL with flow (beyond no flow) followed by decrease at the highest flow rate (Fig. 5f – j). At 2 M, for powers above 20 W, flow can also increase SL beyond no flow and at 50 W all flow rates significantly increase SL beyond no flow (Fig. 5h – j). Thus, by the application of flow, SL can be augmented and maximised for KI systems.

#### 3.4.3 Implications of observed SL trends for phenol and KI concentration and flow

For the phenol systems, without flow, an increase in concentrations (from 0.1 to 20 mM) could increase SL to a point after which (20 to 60 mM) it was generally reduced. A similar trend was previously observed for a 44 kHz plate (high power, un-stabilised) attributed to a reduction in coalescence and increase in quenching respectively. Then, for the KI system, without flow, an increase in KI concentration (from 0.1 to 2 M) generally acted to increase SL. This may be due to salts effects on reducing coalescence/clustering. To confirm de- -coalescence/-clustering effects, more work is suggested to determine the effects of phenol and salt on the bubble population.

The effects of flow on the phenol systems were more complex implying competing effects between changes to bubble characteristics/distributions which augment SL, and those which cause SL quenching to take place. For KI solutions flow could increase SL especially at higher powers. This suggested that flow, may be contributing to the removal of bubbles at the horn tip that would attenuate the sound field. To more accurately determine flow augmentation mechanisms, direct image analysis of bubble behaviour under ultrasound horn conditions is recommended.

## 4 Conclusions

Flow, applied to an ultrasonic horn system, could significantly enhance the degradation of phenol beyond the no flow systems at all applied powers. The maximum increase in degradation, compared to a no flow system was 126% at 24 mL/min and applied power of 10 W where it was comparable or greater than degradation observed at higher powers. Thus, this represents a significant result for increasing the efficiency of such systems and may be particularly applicable to large scale ultrasound treatment reactors.

KI dosimetry and SCL showed similar trends and increases (to phenol degradation), with flow, which suggests degradation followed an oxidation process. Analysis of the intermediates of phenol, and other, degradation products represents further work to confirm this theory for the horn system. Fluid flow could also act to increase SL from water, KI and phenol solutions. Therefore, this work has shown that applied fluid flow to an ultrasonic horn system can significantly enhance both sonochemical and sonoluminescence processes.

The increase in phenol degradation with applied flow could be due to many factors including the removal of attenuating/clustering bubbles from the horn tip region and increasing the transient nature of the bubbles present. The former may reduce attenuation of the sound field and allow more bubbles in solution to be exposed to the field, in turn aiding in the production of radical species for sonochemical activity. The latter may allow for an increase in fragmentation, as the bubbles become more transient in nature, and increase transfer of radical species to solution. To confirm this theory, more work has been suggested to directly image the bubbles present, by such methods as high speed photography, and more accurately determine their characteristics under conditions of applied flow.

## Declaration of Competing Interest

The authors declare that they have no known competing financial interests or personal relationships that could have appeared to influence the work reported in this paper.
